# Supplementary material for: Suidae iPSC-derived macrophages as models for investigating susceptibility and resilience to African swine fever virus
Source: Stem Cell Reports. 2026 Aug 6;21(9):103046. doi: 10.1016/j.stemcr.2026.103046 (PMC13555459; doi:10.1016/j.stemcr.2026.103046)
Supplement: Supplementary file 1 [file mmc1.pdf]

Stem Cell Reports, Volume *21*

Supplemental Information

Suidae iPSC-derived macrophages as models for investigating susceptibility and resilience to African swine fever virus

Tom M. Watson, Lynnette C. Goatley, Stephen Meek, Lel Eory, Sylvia Kohler, Nicholas Berkley, Sofia Sternberg, Melany Jackson, Amy Findlay, Isabella Hoskins, Simon Girling, Joseph Mee, Alan L. Archibald, Finn Grey, Falko Steinbach, Helen Crooke, Christopher L. Netherton, and Tom Burdon

Document S1

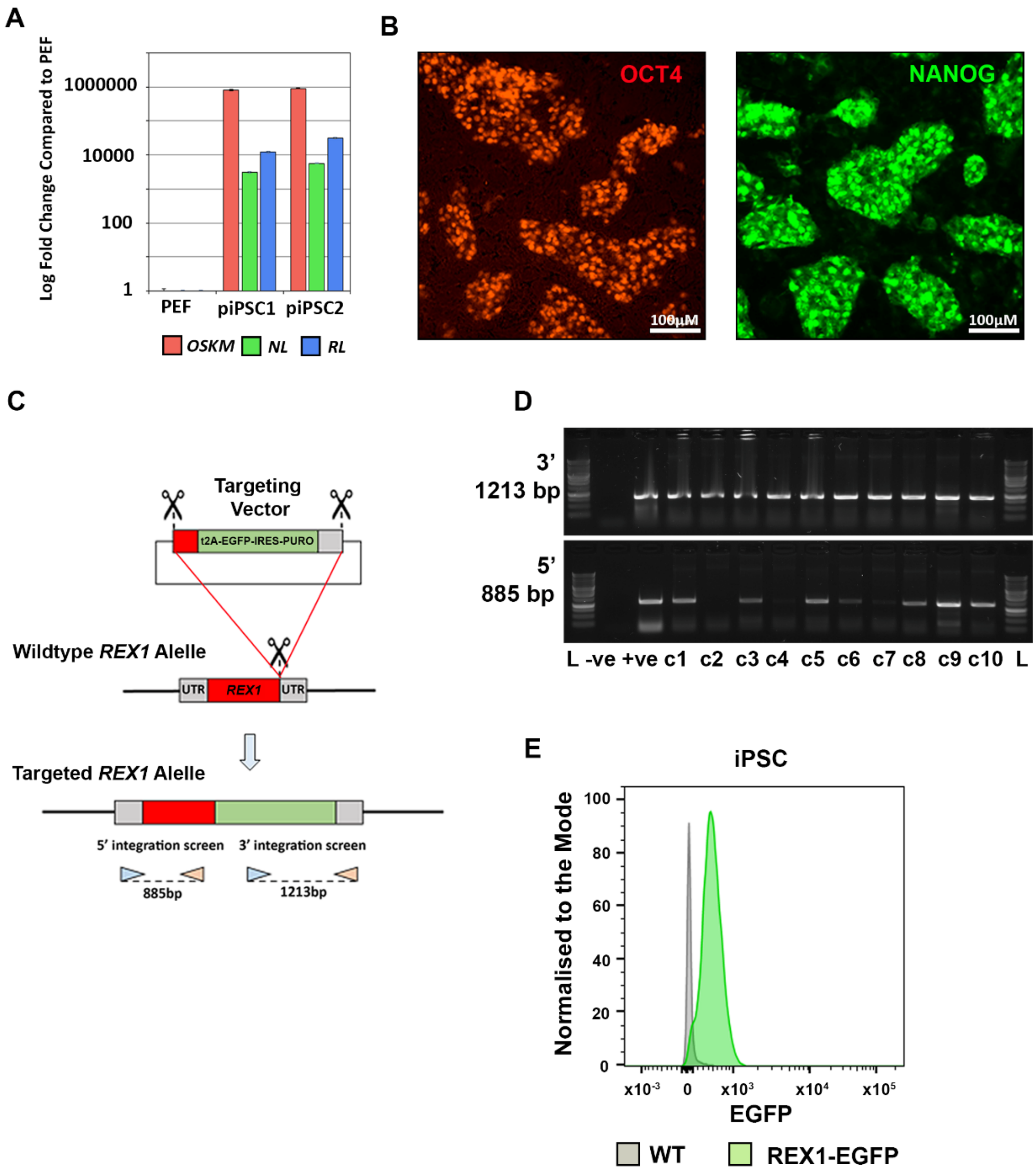

Figure S1 Characterisation of domestic pig iPSC pluripotency factor expression. (A) RT-qPCR expression data showing the log fold change of reprogramming plasmid derived mRNA in piPSC1 and piPSC2 relative to non-transfected fibroblasts. Mean ±SD of three independent experiments. (B) Fluorescent images of OCT4 (Red) and NANOG (Green) protein expression in piPSC1. (C) Diagram showing *REX1-EGFP* Knock-in strategy. CRISPR/Cas9-mediated homology-directed repair, using a repair template (top), allows the generation of the targeted *REX1* allele. PCR product sizes for integration are indicated. (D) PCR genotyping of puro-resistant, EGFP+ clones. Clones 1, 3, 5, 6, 8, 9 and 10 showed the

expected products at both the 5’ and 3’ ends of the integration site. Wild-type, parental, porcine iPSC genomic DNA was used as a negative control (-ve) and the transfected pool as a positive control (+ve). (E) Flow cytometry analysis of porcine *REX1-EGFP* iPSCs and iPSCdMs.

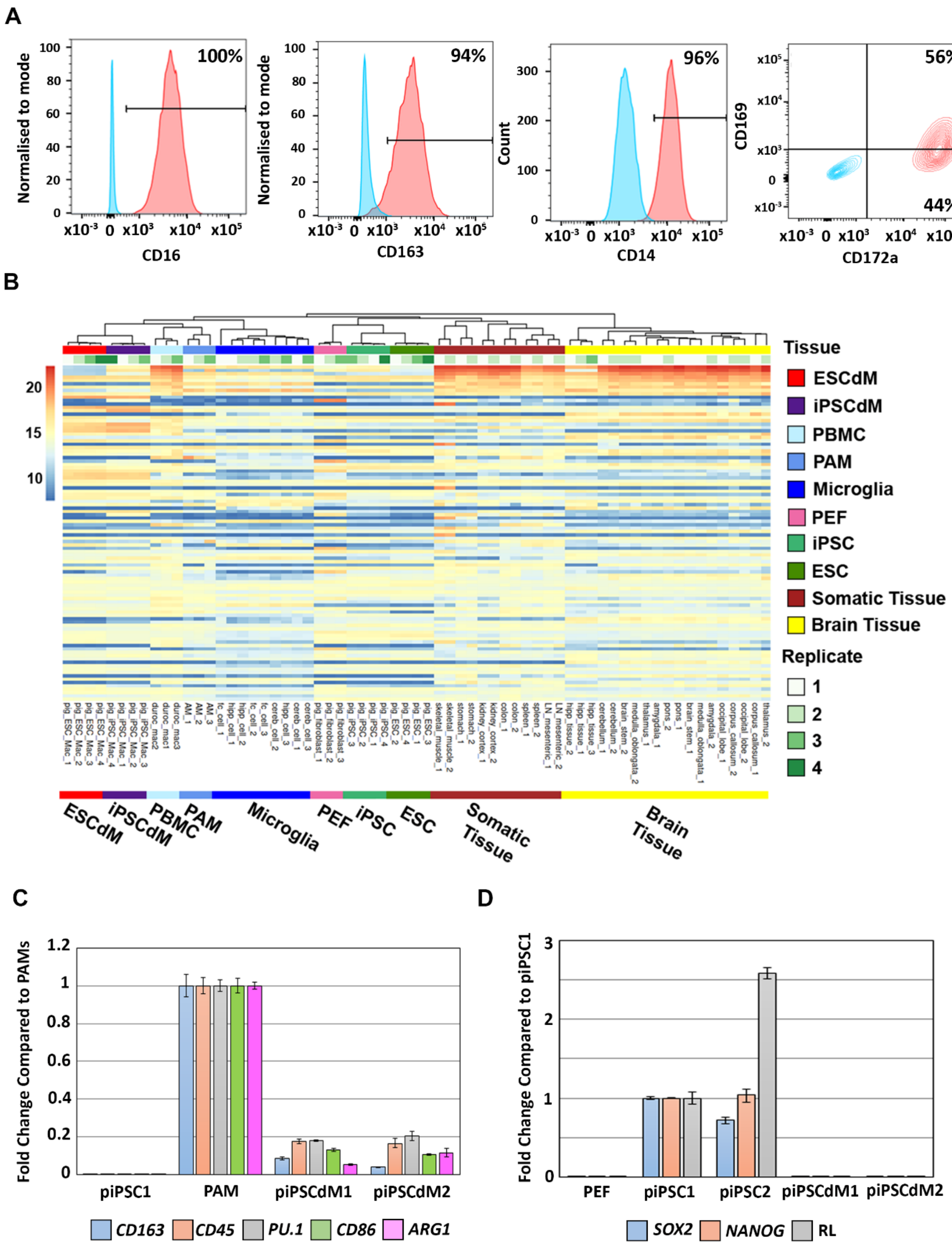

Figure S2 Additional characterisation of piPSC1, piPSC2 and derived iPSCdMs. (A) Flow cytometry of piPSC2 derived Mϕs stained for Mϕ surface markers (Red) relative to isotype controls (Blue). (B) Heatmap showing the hundred most highly expressed genes in cell lines and pig tissues. The tree at the top of the heatmap indicates the hierarchical clustering

between samples. (C) RT-qPCR data showing expression of endogenous Mϕ genes in iPSCdMs compared to undifferentiated piPSCs and *ex-vivo* PAMs. Mean ±SD of 3 independent experiments. (D) RT-qPCR data showing the total (endogenous + exogenous) expression of pluripotency genes *SOX2* and *NANOG*, and the *RARG+LRH1* reprogramming transgene in piPSCs compared to fibroblasts and iPSCdMs. Mean ±SD of 3 independent experiments.

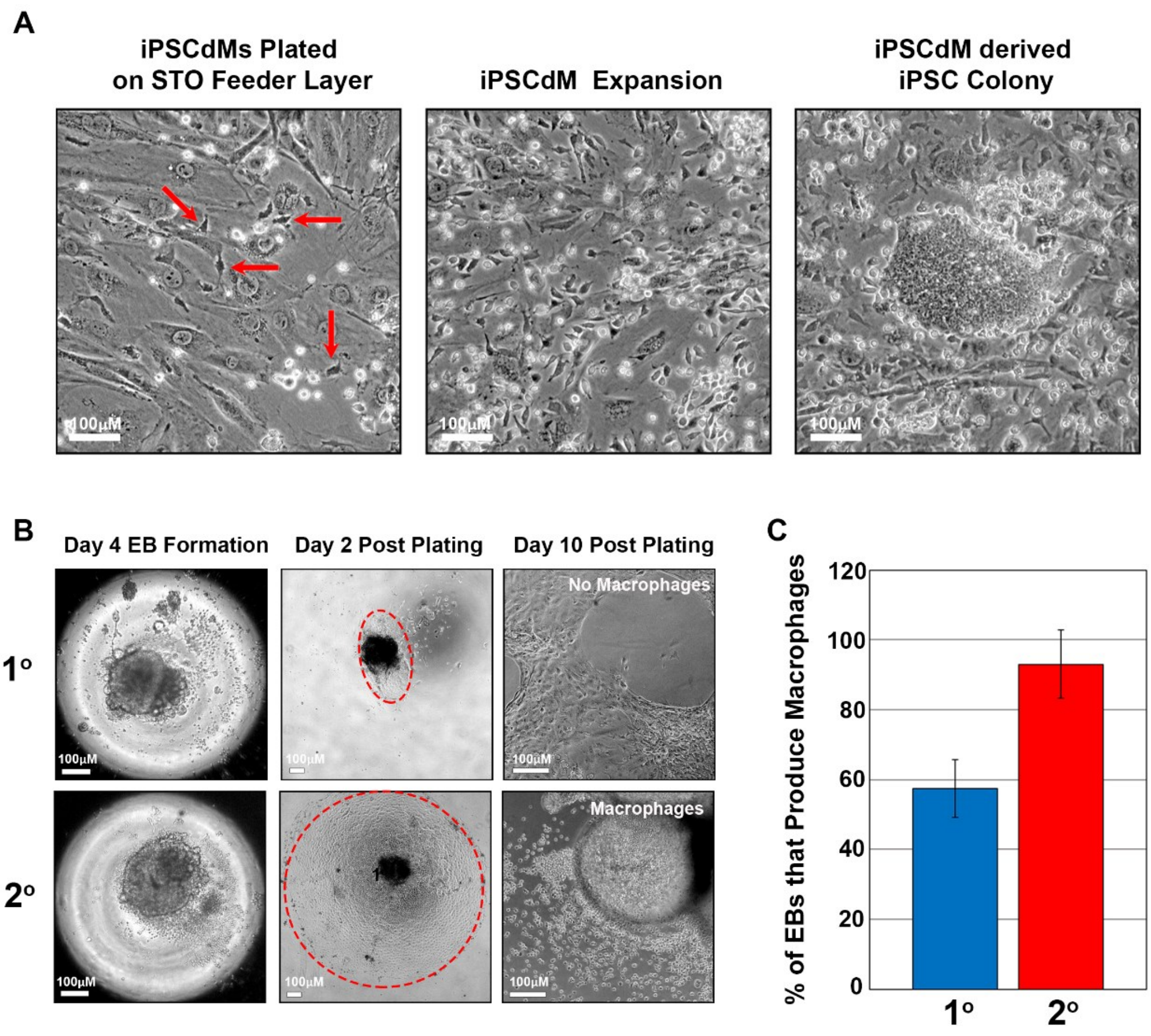

Figure S3 Secondary reprogramming of suid iPSCs to improve Mϕ differentiation potential. (A) Representative bright field images showing the stages of iPSCdM secondary reprogramming. Red arrow indicates iPSCdMs adhered to feeder layer. (B) Representative bright field images showing the effect of secondary reprogramming on EB outgrowth and Mϕ production. (C) Bar graph showing the mean percentage of EBs capable of producing Mϕs, comparing secondary reprogrammed clones (2°) to the parental clone (1°). Mean ±SD between 3 suid iPSC lines (piPSC2, rhiPSC2 and wbiPSC).

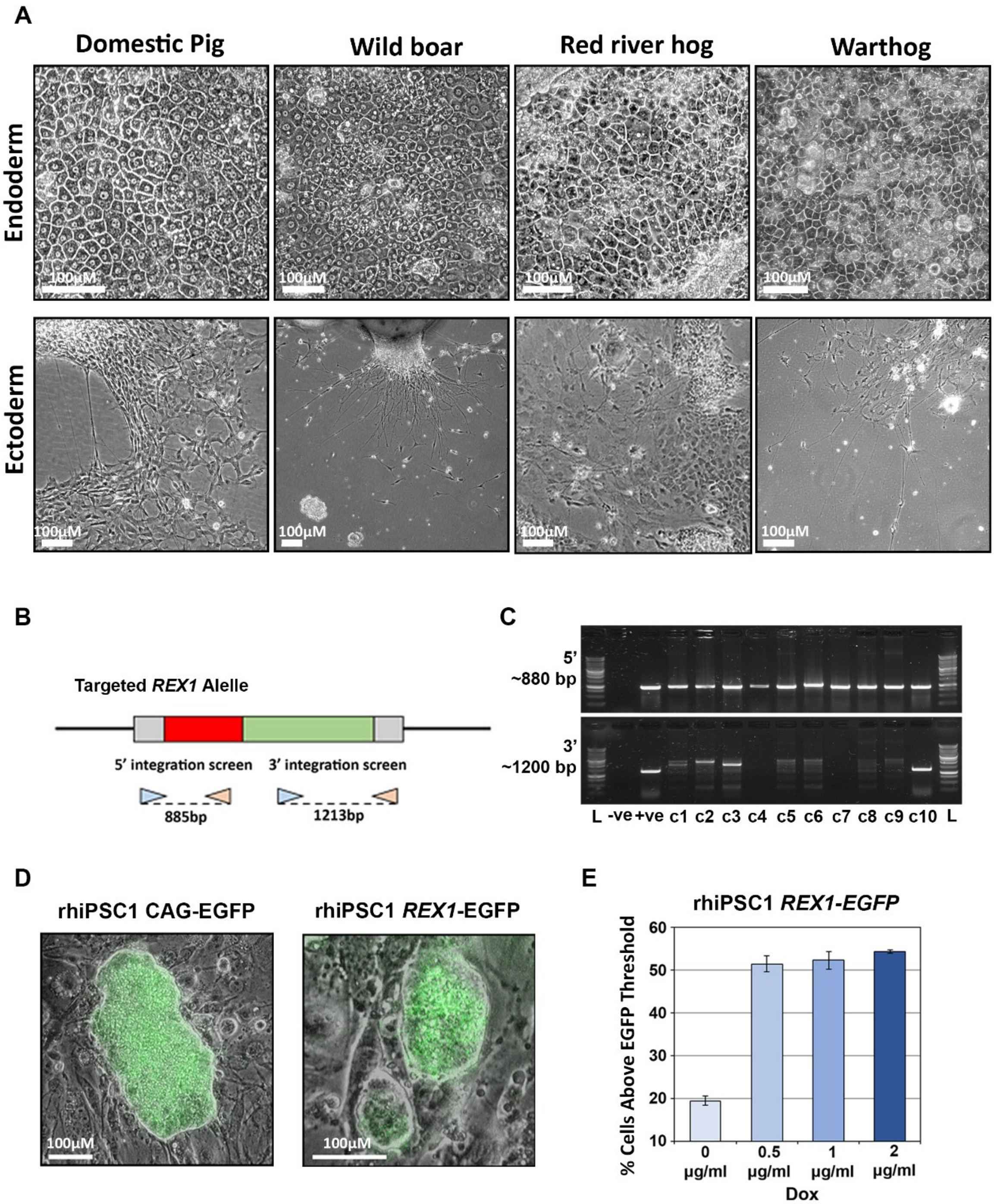

Figure S4 Evidence of multilineage differentiation and gene editing potential of suid iPSCs. (A) Bright field images showing endodermal or ectodermal (neural) morphology in differentiated iPSCs from 4 suid iPSC lines. (B) Diagram showing predicted targeted integration of *REX1-EGFP* construct, and expected product sizes for PCR products identifying correctly integrated construct in rhiPSCs. (C) PCR genotyping of 10 puro-resistant, red river hog EGFP +ve clones. Clones 1, 2, 3 and 10 showed the expected products at both the 5' and

3’ ends of the integration site. The transfected pool was the positive control (+ve) and wild-type and non-transfected RRH genomic DNA the negative controls (-ve). (D) Composite bright field and fluorescent images showing rhiPSCs expressing CAG-EGFP and targeted REX1-EGFP. (E) Bar graph showing a doxycycline (dox) titration in RRH *REX1-EGFP* c10. EGFP analysed by flow cytometry at d4 of titration and threshold set to the first replicate in standard 1 μg/mL dox conditions. Mean ±SD of 3 technical replicates.

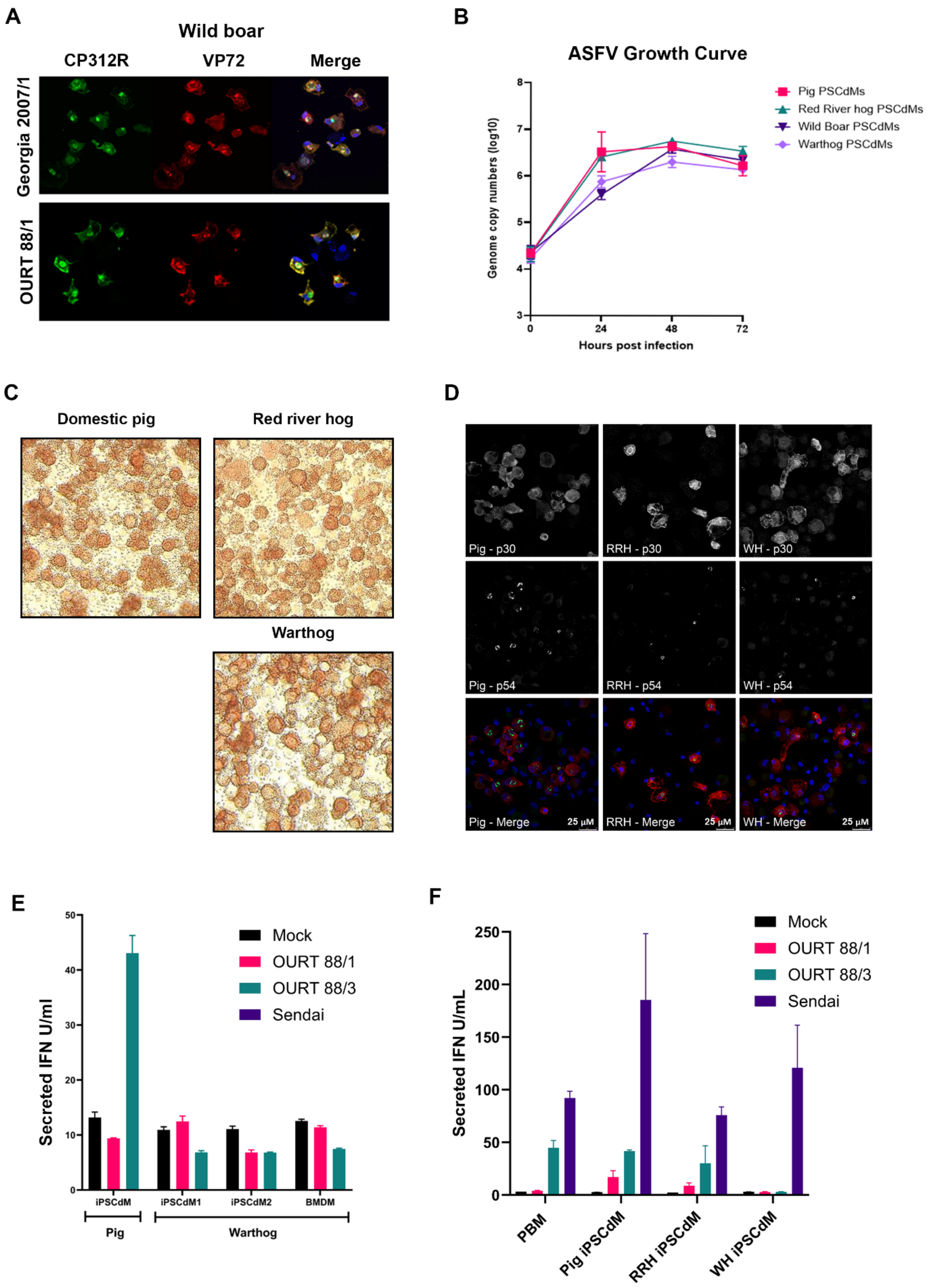

Figure S5 Additional characterisation of ASFV infection in ASF relevant domestic and wild pig iPSCdMs (A) Fluorescent image of ASFV-infected Wild Boar iPSCdMs, 24 hpi, ICC

stained for viral proteins CP312R (green) and VP72 (Red). (B) RT-qPCR analysis of ASFV levels (genome copies) present in supernatants from 4 suid iPSCdMs 24 and 48 hpi with ASFV (Georgia 2007/1). Mean and ±SD of 4 independent experiments. (C) Bright-field image showing haemadsorption in ASFV-infected iPSCdMs 24 hpi. (D) Fluorescent images of ASFV-infected (Attenuated OURT 88/3) iPSCdMs from 3 suid species, 24 hpi, stained for viral proteins p54 (green) and p30 (Red). (E) Production of biologically active IFN from piPSCdM1, whiPSCdM1, whiPSCdM2 and *ex vivo* warthog BMDMs after infection with virulent ASFV (OURT 88/1), attenuated ASFV (OURT 88/3) 24hpi. Mean ±SD of 3 independent experiments. Results interpolated using a universal IFNα standard. (F) Data from Figure 4C with addition of Sendai virus +ve for IFN production.

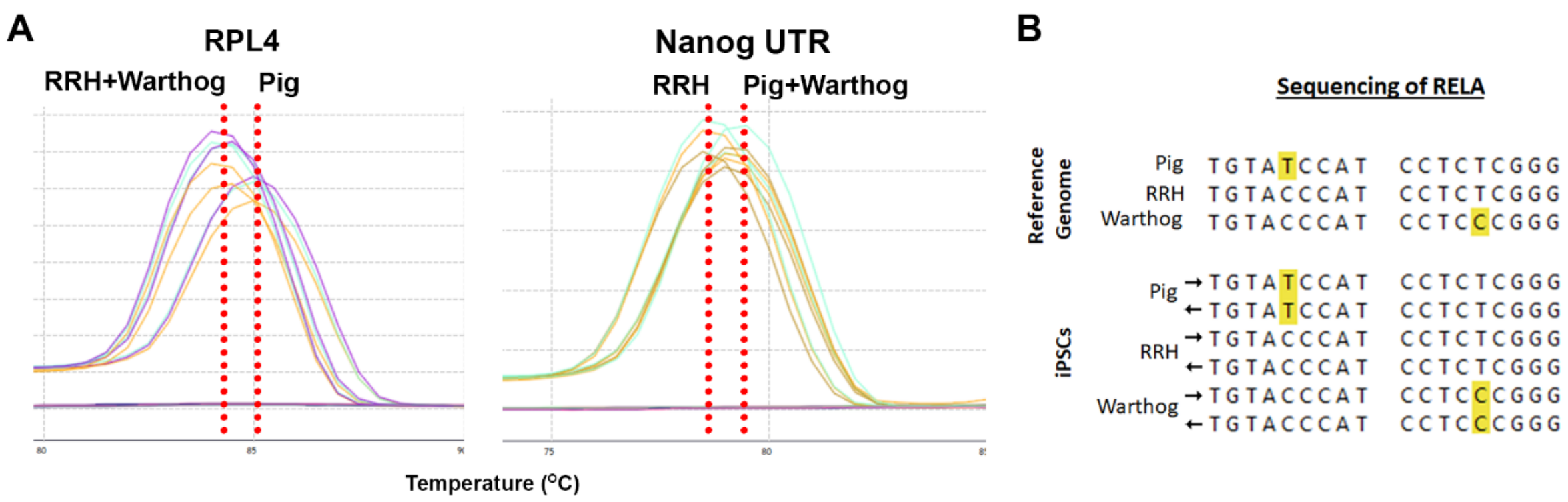

Figure S6 Species identification of suid iPSCs (A) RT-qPCR melt curves using domestic pig specific primers for *RPL4* and *NANOG*. For *RPL4* RRH and warthog have a lower melt curve temperature than domestic pigs. For *NANOG* RRH has a lower melt curve temperature compared to domestic pig and warthog. The combination of the melt curves separates the 3 species. (B) Sequencing of *RELA* in domestic pig, RRH and warthog. Our iPSCs can be identified by previously described SNPs in the *RELA* sequence that match their equivalent reference genome.

Table S1: Oligonucleotide list

qRT-PCR Primers	
**Gene**	**Sequence**
ARG1 For	ACTAGGAAGAAAGAAAAGGCCA
ARG1 Rev	TTCTTCTGGTGTCTTCCCCA
ASFV p72 For	CTGCTCATGGTATCAATCTTATCGA
ASFV p72 Rev	GATACCACAAGATCAGGCCGT
CD163 For	GTGGTCAACTTCGCCTGGTC
CD163 Rev	TCAGGTCCCAGCTGTCATCA
CD45 For	ACCCTGCTCAGAACGGACAA
CD45 Rev	GGGGCACTTGGTGAACTGAC
CD86 For	TGCTTGCTAACTTCAGTCAACC
CD86 Rev	GCTCAGTGGTTGAATTCTTCGT
IFNβ For	GCACTGGCTGGAATGAAACCG
IFNβ Rev	AATGGTCATGTCTCCCCTGGGG
IL-10 For	TAGGGTGTGCCCTATGGTGT
IL-10 Rev	GTGGTTAGGCTTGGAATGGA
IL-12β For	TGAGAAGCAGATGTGACCAGA
IL-12β Rev	TGGAATCACTTTACAGGAAGCA
IL-1β For	TCTGCCCTGTACCCCAACTG
IL-1β Rev	CCCAGGAAGACGGGCTTT
IL-6 For	ACCGGTCTTGTGGAGTTTCA
IL-6 Rev	TTAGGGGTGGTGGCTTTGTC
IRF3 For	TTTTCCCGGCTCACTGTACC
IRF3 Rev	CACACCCCACTTCTCGTCAG
KLF5 For	CCTGAGGACTCATACTGGCG

KLF5 Rev	GGTGATCAGAGCGGGAGAAG
Nanog For	GAATAGCAACAGTGTGATTCA
Nanog Rev	GGAATAGAAGCCCGGGTATT
Nanog UTR For	GGTACCCAGCAGCAAATCAT
Nanog UTR Rev	TTACGGTGCAGCAGAAATTG
NL Plasmid For	TGCACCAGAGTAAGCTGCAC
NL Plasmid Rev	CTCCTTTTGATCTGCGCTTC
OCT4 UTR For	CAAACTGAGGTGCCTGCCCTTC
OCT4 UTR Rev	ATTGAACTTCACCTTCCCTCCAACC
OSKM Plasmid For	TCCAAGCAGAGGAGCAAAAG
OSKM Plasmid Rev	CCAGCCAATTTCAAGAGAGC
PRDM14 For	CCCCAGTGGATGCTTCTCTG
PRDM14 Rev	GGTCTTCCTGGGTGAAGTGG
Pu.1 For	TACAGGCGTGCAAAATGGAA
Pu.1 Rev	AAGTCCCAGTAATGGTCGCT
Rex1 For	TCTGAACCCCTCGTGGAAGA
Rex1 Rev	AGCTTGCTGTAAGCACCTGT
RL Plasmid For	TGCGTGGAGGAAGGAATAAG
RL Plasmid Rev	GCAGAGGAAATGGTCAGGTC
RPL4 For	AATGTCACTTTGCCTGCTGT
RPL4 Rev	CTGGGAATTCGAGCCACAG
RPL4 Species ID For	AGGAGGCTGTTCTGCTTCTG
RPL4 Species ID Rev	TCCAGGGATGTTTCTGAAGG
Sall4 For	CTGCAGATTCATGAGCGCAC
Sall4 Rev	GCAATGGTGTTCTCGATGGC
SOX2 For	GAGCGCCCTGCAGTACAACT
SOX2 Rev	CCCTGCTGCGAGTAGGACAT

SOX2 UTR For	ACGGCCATCAACGGTACACT
SOX2 UTR Rev	TCTCCTCCCATTTCCCTCTTT
TNFα For	GCTCAGCAGTTAGGGACCTG
TNFα Rev	AGCCACATCTGCAACCTACC
**Genomic PCR primers**	
Rex1 5’ screening For	GTTTTCTGAGTACGTGCCAGGC
Rex1 5’ screening Rev	CGGGTCTTGTAGTTGCCGTCGT
Rex1 3’ screening For	TGGGAAGACAATAGCAGGCATG
Rex1 3’ screening Rev	CACACCCCGCCCAACTGCTG
RELA For	GGAAGGGACACTGACAGAGG
RELA Rev	TCAGAAGGGCTGAGAAGTCC
**CRISPR/CAS9 guide**	
Rex1_363	CTTCTTTCACTGATTTGTAT

Table S1 Oligonucleotide list. A list of all the oligonucleotides used in the paper.
